# Supplementary material for: Are carbon emissions trading and green financial instruments synergistic? -Comprehensive quantitative research based on content analysis
Source: PLoS One. 2024 Mar 7;19(3):e0298601. doi: 10.1371/journal.pone.0298601 (PMC10919601; doi:10.1371/journal.pone.0298601)
Supplement: S1 Data — (ZIP) [file pone.0298601.s001.zip › Data/地级市Z_F工作报告.zip!/║■▒▒/╧σ╤⌠╩╨/└┤╫╘╣1⁄2╓┌║┼í╛┬φ┐╦╩2╛▌═°í┐_2014.docx]

各位代表：

　　现在，我代表市政府向大会报告工作，请各位代表审议，并请政协委员和其他列席人员提出意见。

　　一、2013年工作回顾

　　过去的一年，在市委的坚强领导下，全市上下迎难而上，砥砺奋进，全力以赴稳增长、调结构、促改革、惠民生，“两个中心、四个襄阳”建设迈出坚实步伐。

　　（一）主要经济指标持续较快增长，占全省的比重明显提升。1—11月，规模以上工业增加值、固定资产投资、社会消费品零售总额增幅均高于全省平均水平，居民消费价格指数低于全省平均水平；预计全年实现地区生产总值2800亿元以上，同比增长11.5%，规模以上工业增加值、固定资产投资、社会消费品零售总额分别增长14%、30%和14.7%；全年地方公共财政预算收入同比增长37%，增幅居全省第一位；我市主要经济指标占全省的比重进一步提升，省定目标全面完成。县域经济争先进位、竞相发展，7个县（市）区在2012年度全省综合考评中全线进位，襄州区跻身全省10强，枣阳市20强位次前移，老河口市进入全省20强，枣阳市、襄州区分别在全省“三农”考核中居第一、第二名。

　　（二）结构调整扎实推进，发展质量明显提升。工业经济呈现明显的结构性变化。战略性新兴产业突破性发展，1—11月电子信息、新能源汽车、高端装备制造、生物医药、新能源新材料等战略性新兴产业产值同比增长27.2%，占规模以上工业总产值的比重达15%，同比提高3个百分点，我市列入了全国老工业基地调整改造规划，成为首批全国新能源汽车推广应用城市和国家信息消费试点城市；特色产业园区建设扎实推进，优势产业加速集聚，全省重点成长型产业集群达到9个，居全省第二位；优势企业加速成长，新增产销过100亿元的工业企业1家、过50亿元的2家、过10亿元的10家，骆驼集团成为我市首家“中国民营企业500强”，10家企业进入“湖北民营企业100强”。现代服务业快速发展。襄阳北国际物流园、襄阳东农产品物流园、襄阳国际商贸城等市场集聚区开工建设，我市成为全国城市共同配送试点、肉菜追溯体系建设试点城市、中小商贸流通企业服务体系建设试点城市；电商企业新增259家、达到2687家；中国移动湖北公司“四个中心”、华为华中云计算产业园、锦云科技云架构呼叫中心等一批云计算产业项目加快推进，IBM华中卓越云计算中心投入运营；文化旅游产业支撑性项目加快推进，古隆中景区、唐城一期工程等一批重点项目主体完工，新增1个4A级景区，预计全年旅游总收入同比增长20%。现代农业加快发展。新增省级农业产业化龙头企业30家，家庭农场突破1000家，农民专业合作社达到3800家；土地流转面积180万亩，规模经营面积突破100万亩，分别比上年增长一倍；襄州现代农业示范区建设加快推进，以南漳为核心区的有机农业示范区建设扎实起步；农田水利基本建设和高产农田建设力度加大，农业综合生产能力明显增强，百亿斤粮食生产大市地位进一步巩固，我市再次荣获“全国粮食生产先进市”称号，成为全国农业生产全程服务试点、农村公共服务运行机制维护建设试点和中国民生林产业示范区。经济效益明显提升。1—11月，规模以上工业利润、税金同比分别增长29.6%、20.3%；服务业税收占全部税收的比重由上年的35.9%提高到40.9%。

　　（三）投资保持快速增长，新的经济增长点明显增多。实施亿元以上投资项目863个，民间投资和亿元以上项目投资的比重分别达到79.9%、69.8%，拉动全市投资增长27.6和23.5个百分点；实施省市重点项目182个，投资过100亿元、50亿元、10亿元项目分别为8个、10个、96个，竣工投产亿元以上工业项目75个、新增产值750亿元。招商引资成果进一步扩大。实际到位资金1067.7亿元，同比增长37.8%；新注册5个投资过100亿元、8个过50亿元、60个过10亿元的重大项目，华为、中铁建、李尔、伟世通、旭硝子、恩梯恩、考泰斯等7家世界500强企业相继落户，汽车零部件、云计算等产业链招商取得实质性突破，英菲尼迪产业园和东风零部件产业园的产业链集聚效应初步显现。新增国家级示范试点67个，争取项目资金90亿元。

　　（四）统筹城乡力度加大，新型城镇化水平明显提升。新城新区建设和旧城更新同步推进。东津新区完成固定资产投资110.9亿元，两桥两环线控制性工程基本竣工，核心区基础设施实现全覆盖；80万平方米安置房主体完工，十大公共工程有9个开工建设；引进投资过10亿元的产业项目5个，总投资312.7亿元。旧城更新稳步推进，中心城区开工建设“两改”项目27个、竣工2个，开工安置房308万平方米；内环线以内工业企业迁建完成31家、正在转迁36家，专业市场转迁对接基本到位；县（市）新城新区建设全面推进，核心区基础设施基本实现全覆盖，河谷组群发展扎实起步。城市基础性、支撑性功能项目开发系统推进。中心城区城市道路畅通工程成效显著，内环线全线贯通，东津大桥、卧龙大桥、唐白河大桥、胜利街立交桥建成通车，深南大道综合整治以及襄南景观大道等4个出口路建设完工，南北轴线建设快速推进；城市生态景观建设力度加大，新建公园游园10处、绿道112.4公里，新增城市绿地109万平方米；“五城同创”扎实推进，城市管理水平进一步提高；区域性综合交通枢纽功能逐步提升，全市完成交通投资突破100亿元，高速公路和国省干线公路建设快速推进，襄阳至武汉动车每日增至8对，襄阳机场航线增至11条，全年航空旅客、货邮吞吐量分别增长50%和110%。整镇推进新型农村社区建设。以襄城尹集、襄州双沟两个市级试点为示范，10个县级试点有序展开，我市经验在全省推广。生态文明建设扎实推进。节能减排完成省定目标，汉江干流襄阳段各断面水质稳定达标，主城区空气质量优良天数达到314天，城市污水处理厂污泥综合处置示范项目得到联合国开发计划署等国际机构和国家住建部、环保部的高度评价。

　　（五）改革创新持续推进，发展活力明显增强。政府管理创新进一步深化，推进市直部门“大科室制”改革、行政审批“三集中三到位”，行政审批和管理服务事项由251项减少至145项，创新“12345”政府公共服务热线运行机制，促进了行政服务效能提升；建立“三个三分之一”的工作机制，实施路线图管理、台账式管理、绩效管理和流程式、全过程督查，有力推动了重点工作落实；探索市级财政专项资金竞争性分配改革，专项资金使用效益明显提高。金融创新实现新的突破，全市新引进和成立各类金融机构21家，新增融资突破500亿元，银行贷款增幅居全省第一位，市建投公司融资方式由单纯经营土地向经营资产转变；34家企业在武汉股权托管交易中心挂牌，13家企业即将在“新三板”挂牌，27家股权投资机构投资我市企业。土地管理制度创新深入推进，城乡建设用地增减挂钩、低丘缓坡地综合开发利用、城镇低效用地再开发试点取得重大突破。科技创新成效显著，新增2个国家级企业技术中心、20家省级企业技术中心、工程技术研究中心和校企共建研发中心(研究院)，新登记省级以上重大科技成果170项，申请发明专利1900件，新认定高新技术企业69家；以我市企业为主起草19项国家标准，国家标准委批准在襄阳设立了电机软启动技术分会；“隆中对·创业与人才”活动成功举办，引进创新创业团队16个；我市成为国家创新型试点城市、国家可持续发展实验区、国家智慧城市试点，连续五次获得“全国科技进步先进市”称号。对外开放提速。预计全年出口额增长35%，实际外商直接投资增长20%。第三次经济普查取得了阶段性成果。

　　（六）社会建设全面加强，民生保障水平明显提升。民生投入力度加大。民生支出占公共财政预算支出的比重达到73.6%，年初确定的关系民生的“十件实事”全面完成，预计全年城乡居民收入分别增长12%和12.9%。创业就业不断扩大。新增各类市场主体10.5万户、同比增长72.4%，新增微型企业6350家，新增城镇就业10.45万人、农村劳动力转移就业6.2万人。社会保障能力明显提升。城乡居民养老保险制度实现全覆盖，企业退休人员基本养老、城乡最低生活保障、农村五保人员供养以及新农合、城镇职工和居民基本医保等六项保障待遇全面提高，大病保险全面启动，新增机构养老床位1082张；住房公积金制度覆盖城镇就业人员新增1.6万人，全市开工建设各类保障性住房48385套、分配入住16855套，完成扶贫搬迁、农村危房和特困户危房改造11117户。向30多万低收入群众发放了临时价格补贴，向1万名特困残疾人发放了生活补助，帮助5.5万人脱贫，我市扶贫工作在全省考核中蝉联第一；支援琼结、精河、鹤峰发展的各项工作取得了积极成效。社会事业全面进步。学前教育三年行动计划目标全面实现，200所城乡义务教育学校标准化建设全部完成，高中教育质量稳居全省前列，湖北文理学院理工学院新校区和襄阳汽车职业技术学院新校区投入使用；公共文化服务网络逐步完善，我市成为国家公共文化服务体系示范区创建城市；医药卫生体制改革和公共卫生工作继续走在全省前列，基层医疗卫生服务功能进一步增强；人口出生政策符合率高于省定标准，出生人口性别比控制在省定目标以内；全民健身运动深入开展。社会管理创新持续深化，我市成为全国社会管理综合治理优秀市、全国法治城市建设先进单位、法治政府建设和行政复议委员会试点；应急管理、安全生产和食品药品监管力度进一步加大，“三查一促”工作扎实有效，集中排查整改了一批安全隐患，化解了一批不稳定因素。

　　一年来，我们切实加强自身建设，完善工作规则，规范决策程序，促进科学决策；在全国率先清理、规范、公布市县政府部门权力清单，促进行政权力公开透明运行；加强行政执法监督，促进规范执法、文明执法；坚持重大事项向人大报告、与政协协商制度，自觉接受市人大、市政协和社会监督，认真办理人大代表议案、建议和政协建议案、提案；深入开展“假如我是服务对象”大讨论活动，推进市民问政、治庸问责常态化，有力促进了政府各部门作风转变、效能提升；认真履行廉政建设责任制，严肃查处违法违纪案件，严格执行中央八项规定，大幅压缩“三公”经费支出，加强政府投资项目和财政资金监管，规范公共资源配置交易，促进了廉洁政府建设。

　　各位代表，过去一年的成绩来之不易，这是市委带领全市广大干部群众认真贯彻省委、省政府决策部署，团结奋进、攻坚克难、改革创新的结果。在此，我代表市人民政府，向全市人民，向所有纳税人，向各位人大代表、政协委员，向各民主党派、工商联、人民团体和无党派人士，向驻襄人民解放军、武警官兵和公安干警，向所有关心支持襄阳发展的同志们、朋友们，表示衷心的感谢，致以崇高的敬意！

　　回顾总结过去一年的工作，我们清醒地看到，经济社会发展还面临许多现实困难和挑战。主要表现在：经济总量不大与结构不优并存，做大总量、推进经济转型升级的任务十分艰巨；“三农”基础脆弱与城镇化质量不高并存，推进新型城镇化和城乡发展一体化的任务十分艰巨；发展环境不优与营商成本过高并存，全面深化改革、转变政府职能、优化营商环境的任务十分艰巨；社会治理压力加大与民生保障能力不足并存，扩大公共产品供给、创新社会治理方式、增强公共安全保障能力的任务十分艰巨。对这些问题,我们将在今后的工作中努力加以解决。

　　二、2014年工作安排

　　党的十八届三中全会发出了全面深化改革的动员令，改革开放到了一个新的重要关头，对各级政府是一次重大考验。当前，宏观经济环境依然复杂严峻，我国经济面临很多挑战，我市经济保持持续增长的压力很大；但更要看到，我们正面临着许多历史性发展机遇。随着中央推进新一轮改革的举措陆续出台，中央经济工作会和新型城镇化工作会确定的一系列政策措施逐步落实，必将进一步激发经济发展的内生动力和市场活力；长江经济带上升为国家战略，汉江生态经济带上升为省级战略，汉江流域开放开发有望融入长江经济带建设，给襄阳带来了重大发展机遇。从我市发展条件看，多年积聚的能量加速释放，随着一批支撑性、功能性重大基础设施项目规划确定、陆续开工、相继建成，一批重大产业项目进入收获期，一批优质企业快速壮大，跨越发展的支撑明显强化；多项国家级试点示范落户襄阳，全国老工业基地调整改造直接惠及襄阳，资源集聚能力和城市影响力明显提升；一系列促进经济增长的改革创新举措效应持续显现，市场信心和发展动力明显增强。面对新的发展形势，我们必须清醒认识面临的挑战，切实增强机遇意识、责任意识、使命意识，坚持底线思维，以更大的智慧和勇气大胆改革、锐意创新，牢牢把握新一轮改革发展的主动权。

　　今年政府工作的总体要求是：深入贯彻落实党的十八届三中全会、中央经济工作会、省委十届四次全会和市委十二届八次全会精神，按照稳中求进、又好又快的总要求，坚持改革统领，着力增强发展的内生动力和市场活力；坚持创新驱动，着力推动经济转型升级；坚持“四化同步”，着力推进新型城镇化和城乡发展一体化；坚持民生优先，着力加强社会建设、增进人民福祉；坚持绿色发展，着力加强生态文明建设，加快建设省域副中心城市和汉江流域中心城市，努力在全省“建成支点、走在前列”大局中谋求新作为、创造新业绩。

　　今年经济社会发展的主要预期目标是：地区生产总值增长12%，规模以上工业增加值增长15%，全社会固定资产投资增长25%，社会消费品零售总额增长15%，出口和实际外商直接投资分别增长12%、15%，地方公共财政预算收入增长20%，城乡居民收入分别增长12%，全面完成省政府下达的物价调控、计划生育、节能减排、安全生产等约束性指标。围绕实现上述目标，重点做好七个方面的工作：

　　（一）以强化投资拉动经济增量提质

　　着力提高招商引资质量。完善招商引资考核机制，进一步创新招商引资方式，深入推进专业招商、产业链招商，培育壮大产业集群；大力引进跨国公司地区总部、财务公司、研发中心等功能性机构，积极引进新兴服务业态。力争全年招商引资到位资金突破1450亿元，引进投资过5亿元的项目100个、过10亿元的65个、过50亿元的10个、过100亿元的5个。

　　强力推进项目建设提速增效。严格重点项目路线图管理，强化项目秘书制、全程督查制，实行集中竣工投产制，全力推动项目快开工、早见效。全年实施900个亿元以上项目，投资过100亿元、50亿元的项目分别达到10个、15个，力争有200个亿元以上项目竣工。突出抓好英菲尼迪国产化、东风零部件产业园、东风井关农机装备产业园、际华襄阳纺织工业园等525个亿元以上工业项目建设，力争有170个项目竣工投产，工业投资占全市固定资产投资的比重达到60%以上。加快区域性综合交通枢纽建设，确保襄阳机场新航站楼主体工程开工、停机坪扩建完工，争取蒙西至华中铁路煤运通道和武襄十城际铁路襄阳段开工，促进谷竹高速襄阳段建成通车，续建新建15条一、二级公路，加快建设襄阳新港，扎实做好郑万高铁、雅口航运枢纽等一批重大项目前期工作。加快重大能源项目建设，开工建设新集水电、大唐热电、华润安能热电等项目，加快华电三期、老河口电厂等项目前期工作。

　　全力争取国家政策和投资支持。抢抓国家推进重点领域改革的重大机遇，力争一批改革试点落户襄阳先行先试；抢抓国家建设长江经济带的机遇，争取将汉江经济带融入其中；抢抓国家支持军民融合发展的机遇，依托国家军民结合产业基地，争取军工企业总部将更多的项目布局襄阳；抢抓国家投资政策调整的机遇，积极策划争取一批国家和省投资项目，努力在争取外国政府贷款上实现更大突破。

　　（二）以创新驱动推动经济转型发展

　　加快打造万亿工业强市。深入实施主导产业转型升级三年行动计划，推进优势产业集聚集群发展。以产业链垂直整合推动产业集聚。大力推动我市产业龙头企业、领军企业和上市公司整合市内外关联企业，带动产业集聚；巩固提升汽车产业龙头地位，重点在引进乘用车、新能源汽车、专用车、农用机械等整车项目上突破，在吸引国内外核心和关键零部件企业集聚上突破；大力实施传统产业转型升级计划，推动食品、纺织、化工等产业技术改造、品牌培育、链条延伸、业态创新；大力实施战略性新兴产业倍增计划和高新技术产业链技术创新规划，重点培育智能制造为主的高端装备制造、云计算为主的电子信息、纯电动和混合动力为主的新能源汽车等优势新兴产业，大力发展新能源新材料、航空航天、生物医药、节能环保等特色新兴产业。以培育优势企业带动产业集聚。大力实施百家龙头骨干企业成长工程、百家高成长性中小企业星火工程、百家企业技术改造示范工程，深入实施促进小微企业发展三年行动计划，力争全年新增1家产值过100亿元、2家过50亿元、10家过10亿元、50家过亿元的骨干企业，新发展小微企业6000家以上，争创中国驰名商标3件以上。以特色产业园区建设推动产业集聚。健全“五个一”的推进机制，创新特色产业园区建设运营机制。以推动服务业与制造业融合促进产业集聚。大力引进和培育研发设计、成果转化、检验检测、专业物流、产业投融资等生产性服务业态，规划建设检验检测园区，着力打造区域性检验检测中心；大力推广云制造服务和工业互联网技术运用，加快推进“两化”融合。

　　加快发展壮大现代服务业。大力推进区域性市场枢纽建设，着力打造长江中游商业功能区的重要支撑。以商圈建设引领城市商业业态升级，加快建设东津新区区域级核心商圈、人民广场市级核心商圈、长虹路等城市副商圈，引进一批国内外知名商家、一线品牌、先进业态，规划建设1—2个夜消费特色街区。以优化消费环境激活消费需求，全面落实鼓励消费的信贷、财税政策，扩大汽车、住房等大宗消费，培植信息、健康、养老、文化、旅游和社区服务等消费热点，促进消费升级。以创建国家电子商务示范城市推动电商产业突破性发展，着力打造区域性电商产业基地。以发展专业物流为重点建设现代物流体系，加快建设襄阳东农产品物流园、襄阳北国际物流园、襄阳西综合物流园和临港物流园，促进物流业集中集聚、有序发展。以建设“襄阳云谷”带动信息服务业跨越式发展，大力推进华为、IBM、中国移动、锦云科技等云计算产业项目建设，加大云服务推广应用力度，积极发展软件和信息服务外包，加快建设“智慧城市”。以发展总部经济提升服务业层级，规划建设2—3个总部经济集聚区。以“一城两文化”引领文化旅游业融合发展，加快建设襄阳古城、古隆中、岘山、鱼梁洲等文化旅游区，开工建设米芾文化创意产业园、文化传媒产业园和文化科技产业园，推动一批县（市）重大文化旅游项目建成运营，实施城市旅游标准化建设，加强旅游策划营销，推进区域旅游合作，提升襄阳文化旅游的吸引力。

　　加快实施创新驱动发展战略。以平台建设提升创新能力。着力推动国家和省级科技孵化器特色化、专业化、集约化发展，力争创建省级以上研发平台10个。以协同创新突破关键技术。整合政府、企业、高校、科研院所等多方面创新资源，共同组建科研公司或研究院，支持支柱产业的关联企业组建产业技术创新联盟，重点建设10个产学研合作创新示范基地，支持优势企业积极参与和牵头制定国家标准。以体制机制创新集聚创新资源。创新科技投入机制，强化财政投入导向性作用，建立政府创投基金对社会股东和管理团队让利的机制，争创国家科技与金融结合试点市，激励企业加大研发投入，吸引各类资本投入科技型企业；充分发挥国家创新型试点城市等“国牌”优势，争取国家创新资源和国内外高端创新资源布局襄阳；创新人才集聚机制，深入实施隆中人才支持计划等重大人才工程，积极创建国家级“海智计划”工作基地，力争在引进国内外科技领军人才和高层次创新创业团队上实现新的突破。

　　（三）以构建新型农业经营体系推进现代农业强市建设

　　加快构建新型农业经营体系。大力培育新型农业生产经营主体，力争家庭农场发展到1500家，专业合作社发展到4500家。建立完善农村承包地流转和仲裁平台，引导农村承包地规范流转，发展多种形式规模经营，全年新增土地流转面积60万亩、规模经营面积20万亩。以开展全国农业生产全程服务试点为契机，每个县（市）区创办一个试点，建立“管理在县、服务在乡”的机制，组建跨区域的农业综合服务机构，鼓励社会力量兴办各类农业社会化服务组织。以开展全国农村公共服务运行维护机制建设试点为契机，完善“以奖代补、先做后补”的农村“以钱养事”机制，着力解决村级公共服务最后一公里的问题。着力提高农业综合生产能力。以实施鄂北粮食增产工程为重点，加快推进以鄂北水资源配置工程、水库除险加固工程、小农水工程等为重点的农田水利基本建设，大力推进土地整治，整市推进高产示范创建，夯实百亿斤粮食生产大市基础。强化农业科技推广，建立龙头企业牵头、高校和科研院所协同、新型农业经营主体参与的农业科技推广机制，大力推进科技入户。

　　大力发展生态友好型农业。整合南漳、谷城、保康、老河口生态农业资源，规划建设“中国有机谷”，着力打造绿色安全食品生产示范区；支持襄州、枣阳、宜城创建国家级现代农业示范区；以发展生态、观光农业为重点，建设樊城都市农业示范区；推进玫瑰、牡丹、核桃、油茶、食用菌、中药材为主的特色林产品产业化、规模化，积极发展生物质能源林和用材林基地，加快建设中国民生林产业示范区。深入推进“两清、两减、两治”为重点的农业生态建设，大力发展高产、优质、高效、生态、安全农产品，力争全年新认证有机、绿色、无公害食品40个以上。

　　突破性发展农产品加工业。大力实施农产品加工业三年倍增计划，优化农产品加工园区布局，引导农产品加工业集聚集群集约发展，着力培育在全国有影响的特色农产品加工集群；大力支持骨干龙头企业通过资产重组、资源整合和产业链重构，组建农产品加工企业集团，大力引进行业龙头企业，积极培育农产品品牌，力争新增1—2家国家级龙头企业。

　　（四）以新型城镇化带动城乡发展一体化

　　坚持以人为本，着力推进以人为核心的城镇化。促进有能力在城镇稳定就业和生活的常住人口有序实现市民化，把让群众生活更舒适的理念融入城镇规划建设的血脉中、体现在城镇化过程的每一个细节中。坚持规划引领，按照生产空间集约高效、生活空间宜居适度、生态空间山清水秀的总体要求，科学编制、严格执行新型城镇化发展规划，确保一张蓝图干到底，保证规划的权威性、严肃性和延续性，促进中心城市、卫星城市、特色镇、新型农村社区四位一体、有序推进、功能互补、协同发展。坚持因地制宜，一切从实际出发，分类指导，不搞一哄而上，不搞一刀切，不搞统一模式，不搞大拆大建，不强迫农民“上楼”。

　　坚持双核带动，着力推进新城新区建设和旧城更新。统筹推进东津新区基础设施建设和主导产业培育。实现70平方公里起步区基础设施全覆盖，确保安置房有100万平方米主体完工、150万平方米开工建设，市民中心、技师学院一期交付使用，科技馆、图书馆、文化艺术中心、医疗中心、优质高中等主体工程完工，加快推进大剧院、会展中心建设；确保区域性商业中心和机器人产业园、文化创意产业园、华科工研院开工建设，华为云计算产业园投入运营。启动建设庞公新区、樊西新区。有序推进旧城更新。合理安排中心城区“两改两迁”项目建设时序，建成安置房308.5万平方米、入住居民10万人。坚持城区经济和县域经济双轮驱动，促进区域协调发展。积极推动老河口、谷城与丹江口组群发展，在汉江生态经济带开放开发中先行先试；推动襄宜南一体化发展，支持枣阳中心城区与吴店、兴隆两个城镇组团发展，促进保康生态旅游试验区建设，大力支持县域经济争先进位。扎实做好支援琼结、精河、鹤峰发展的各项工作。

　　坚持基础先行，着力推进城市功能开发。着力提升城市交通功能。加快建设中心城区“两轴三环九放射”骨干路网体系，确保南北轴线贯通，启动东西轴线建设，大力推进中环线和外环东线建设，建成园林大道北出口等出口路，新建、改建主次干道79条；大力发展公共交通，建设城市步行和自行车交通系统。加强市政配套设施建设。实施地下综合管廊工程，新增88公里供水管网，实施污水治理项目18个，改造续建配套泵站10座，建设截污干管55公里，新建改建排水管网155公里，延伸燃气管网77公里。

　　坚持文化传承，着力打造山水园林名城。以强烈的文化意识指导城镇规划建设和管理，将襄阳优秀的特色文化元素融入城镇规划，着力培育有历史记忆、地域特色的美丽城镇。加大历史文化名城、名镇、名村的保护力度，实施襄阳古城修复工程，推进陈老巷等历史文化街区和会馆的保护与改造，完善城市标识系统，征集推介襄阳十景，延续城市历史文脉。着力培育生态园林景观，大力实施中心城区清水入城和汉江岸线、城市道路景观建设工程，加快推进城市公园、游园、植物园建设和绿地改造升级，新增绿地100万平方米；加大城市山水生态资源的保护力度，尽可能减少对自然的干扰和损害，让城市融入大自然，让居民望得见山、看得见水、记得住乡愁。大力推进“五城同创”，力争成功创建国家森林城市、通过国家卫生城市考核验收。

　　坚持整镇推进，着力建设美丽乡村。坚持全域规划、镇村联动，将10个新型农村社区建设试点镇作为“四化同步”发展试点，统筹推进特色镇、新社区和美丽乡村建设。因地制宜培育特色镇，加强基础设施建设，发展壮大镇域经济，强化产业支撑。整镇推进新型农村社区建设，以开展城乡建设用地增减挂钩试点为切入点，引进市场主体有序推进新型农村社区试点建设，创新集体资产运营管理机制和新型农村社区管理机制。深入推进村庄整治，完善城乡垃圾清运处理体系；实施绿色示范乡村工程，打造一批高标准绿色示范村；加强古村落原始风貌的保护，慎砍树、不填湖、少拆房，在原有村庄形态上改善居民生活条件。

　　坚持机制创新，着力增强新型城镇化发展的动力活力。建立多元化可持续的城镇化投融资机制。吸引社会资本参与城市公用设施投资运营，加快市、县（市）投融资平台体制机制创新，支持特色镇组建专业化投融资公司，利用资本市场进行股权、债券融资，拓宽融资渠道。创新人口管理方式。探索农业转移人口市民化成本负担机制，建立城乡统一的居民登记制度和无门槛的居住证制度，有序放开中等城市落户条件，全面放开建制镇和小城市落户限制，稳步推进城镇基本公共服务常住人口全覆盖，提高居民生活质量。

　　（五）以重点领域和关键环节改革增强发展的内生动力

　　深化行政体制改革。加快转变政府职能。推进简政放权，减少行政审批事项，实施审批流程再造，在高新区、经开区试行行政审批“园内办结”；改革工商登记制度，实行市场主体登记一表制申报、先照后证，放宽住所登记条件，允许小微企业一地多照、一照多地；理顺市、区事权关系和支出责任；推进行政执法体制改革；创新公共产品提供方式。优化政府组织结构。深入推进市、县（市）区政府机构改革，积极稳妥推进“大部门制”改革，稳步推进“区园办（镇）一体化”管理体制改革，深化文化、医药卫生和食品药品监管体制改革，加快事业单位分类改革。创新编制管理，严格控制机构编制。

　　促进多种所有制经济竞相发展。深化国有企业改革。完善国有资产管理体制，以管资本为主加强国有资产监管，以股权多元化、经营集团化为重点推进政府投融资和担保类企业改革，以坚持国有控股、实现股权多元为重点推进公用类企业改革，加快解决国有企业改制遗留问题。大力发展混合所有制经济。鼓励非公有资本、集体资本参与国有企业改革，大力发展交叉持股、相互融合的混合所有制经济，鼓励发展非公有资本控股的混合所有制企业。鼓励非公有制资本进入特许经营领域和国家放开的垄断行业，实施“个转企”和领军私营企业培育工程，促进私营企业做大做强。

　　积极推进土地管理制度创新。探索建设用地新机制。坚持整镇推进城乡建设用地增减挂钩试点，探索建立城乡建设用地增减挂钩指标储备制度和复垦结余土地指标交易制度，逐步建立增减挂钩指标、建设用地指标以及耕地占补指标交易有形市场，实行有偿使用，分类管理；大力推进低丘缓坡地开发利用和城镇低效用地再开发试点，盘活存量建设用地。加快构建城乡统一的建设用地市场。加强集体土地所有权登记发证工作，稳步推进农村集体建设用地和宅基地使用权确权登记发证，启动农村土地承包经营权确权登记发证；加快建立农村集体经营性建设用地流转制度和价格评估体系，明确有偿使用方式和收益分配比例；依托国有建设用地交易市场，建立集体经营性建设用地流转平台。

　　大力推进财政金融改革创新。深化财政管理体制机制改革，实施全面规范、公开透明的财政预算制度，推进财政预算资金全过程绩效管理，深化财政专项资金竞争性分配改革，建立规范合理的政府债务管理和预警机制。大力推进金融创新，千方百计扩大融资渠道，新增社会融资突破600亿元；力争引进银行、证券、保险等各类金融机构10家以上，组建民营银行、地方法人银行取得实质性进展，发展小微企业金融服务机构实现新的突破；大力发展融资租赁、直接债务融资、保险融资，设立小微企业集合信托（私募）债权基金，实现县（市）区债务融资工具运用全覆盖，探索发展互联网金融，推进资产证券化；力争有1家企业在主板上市、10家企业在“新三板”挂牌、30家企业在武汉股权托管中心挂牌、引入20家股权投资机构，探索设立油脂交易所、城市矿产交易所、林业产权交易中心等特色产权交易市场；着力优化金融环境，实施企业信用提升工程，建立公众数据信用平台和公众金融服务中心，构建面向广大市民的电子支付体系；规范民间金融管理，加强风险预警和处置。

　　加快构建开放型经济体系。对接国际先进理念和规则，着力营造稳定、透明、可预期、法治化的营商环境，力争利用外资实现更大突破。支持优势企业到境外投资，参与国际竞争；大力引进沿海加工贸易产业链，着力建设和培育出口基地；着力建设国际物流大通道，大力推进无水港建设、襄阳机场改扩建和一类航空口岸申报，规划新建铁路货运中心，争取实现襄阳至宁波五定班列常态化，加快建设渝新欧中南地区货物集结和分拨运营基地，推动与郑新欧、汉新欧等欧亚大陆桥连接贯通。

　　（六）以绿色低碳清洁发展建设生态文明

　　强化自然生态系统和环境保护。严守生态保护红线，严格执行主体功能区规划，开展“绿满襄阳”行动，申报一批国家自然保护区和湿地公园，加强水土流失治理，推进河流、水库生态修复和水系连通。深入实施蓝天碧水工程三年行动计划，加强大气污染源头治理，建立健全城市空气质量发布体系和重点污染源数据传输与监控机制，强力推进重点行业和企业脱硫脱硝除尘改造，加强城市扬尘和机动车尾气污染控制，加大农作物秸秆禁烧监管和综合利用力度，确保全年空气质量优良天数保持在310天以上，努力让广大居民呼吸新鲜的空气；加强水环境保护和治理，认真落实汉江保护目标管理责任制，确保汉江干流襄阳段水质稳定在现有水平，让广大居民喝上干净的水。积极创建全国生态文明先行示范区。

　　强力推进节能减排。扎实开展低碳城市试点，实施绿色建筑行动，加快推进东津新区绿色示范城区建设；推动企业节能改造和清洁生产改造；积极发展绿色交通，推广使用各类新能源汽车1260辆。狠抓主要污染物减排工作，强力推进城镇污水厂及配套管网建设、农村面源污染治理、规模化畜禽养殖污染治理。大力发展循环经济，依托国家“城市矿产”示范基地和国家级区域性再生资源利用示范基地，发展再生资源利用产业，支持谷城国家级循环经济示范县建设，支持河谷组群共建循环经济示范园区；加快推进城市污水处理厂污泥、餐厨废弃物资源化利用和无害化处理项目建设。

　　加强生态文明制度建设。坚决执行最严格的耕地保护制度，建立供地率、地耗率、闲置低效用地处置率与建设用地指标分配挂钩机制，促进集约节约用地。建立政府调控与市场运作相结合的矿产资源配置机制，提高资源利用效率。认真落实最严格的水资源管理制度，推进节水型社会建设。严格执行主要污染物总量控制、水环境影响评价、建设项目“三同时”、限期治理、挂牌督办、环保后督查等制度，完善环境有偿使用、排污费征收等经济政策，健全生态环境保护责任追究制和环境损害赔偿制度。

　　（七）以保障和改善民生为重点加强社会建设

　　努力为人民群众提供更多的增收致富机会。大力实施居民收入倍增计划。深入推进创业就业富民工程，抓好全国技能振兴示范市建设，着力打造“中国技工之乡”；全面落实鼓励和扶持创业就业的优惠政策，大力推进全民创业，实施大学生创业就业行动计划。探索增加农民财产性收入的渠道，赋予农民对集体资产股份占有、收益、有偿退出及抵押、担保、继承权，保障农户宅基地用益物权，深化集体林权制度配套改革；在保障农民住有所居的前提下，探索建立宅基地有偿退出机制。完善城镇居民收入增长促进机制，合理调整最低工资标准，全面推行企业工资集体协商制度，完善低收入群体物价补贴联动机制。

　　努力为人民群众提供更高水平的社会保障。加强社会保险扩面征缴，积极推进社会保障全覆盖。实施城乡居民养老保险制度衔接和转移接续，完善被征地农民社会保障机制。加快建立城乡一体的医疗保障体系，推进各类基本医疗保险制度有效对接，认真落实重特大疾病医疗保险和救助制度。建立健全社会救助体系，完善城乡最低生活保障制度，建立农村“五保”供养金自然增长机制。完善住房保障制度，积极推进住房公积金制度全覆盖，加快建设各类保障性住房。

　　努力为人民群众提供更优质的公共服务。努力办好人民满意的教育。推进学前教育普惠发展，积极创建学前教育示范县；推进城乡义务教育资源均衡配置，进一步改善教学条件，实行义务教育学校校长、教师轮岗交流；加快建设优质高中，进一步提升高中教育整体质量和水平；充分发挥职教联盟的作用，加快培养技能型人才；大力支持湖北文理学院创建综合性大学、襄阳职业技术学院创建四年制高等职业院校。着力提高人民健康水平。启动第二批县级公立医院改革试点，支持社会力量举办非营利性医疗机构、参与公立医院改制重组，积极组建医疗联合体，加强基层医疗卫生机构和中医药服务体系建设；坚持计划生育基本国策，严格执行人口与计划生育政策；加强城乡体育设施建设，促进全民健身运动深入开展。大力发展文化事业。加快推进市县乡村四级公共文化设施建设，深入实施文化惠民工程，促进基本公共文化服务标准化、均等化，积极创建国家公共文化服务体系示范区。

　　努力为人民群众营造更和谐的社会环境。创新社会治理。改进社会治理方式，提高社会治理水平。积极推进法治城市建设，切实加强依法治理。稳步推进中心城区大社区改革试点，增强社区公共服务功能。大力培育各类社会组织，发展壮大志愿者队伍，鼓励社会力量共同参与社会治理。创新有效预防和化解社会矛盾体制机制，深入实施“平安五小工程”，坚持群众工作“三事工作法”，完善人民调解、行政调解、司法调解联动机制，加强社会稳定风险评估；深入推进信访工作法治化，扎实开展行政复议委员会试点，积极开展“律师进社区”活动。高度重视食品药品安全，用最严谨的标准、最严格的监管、最严厉的处罚、最严肃的问责，确保人民群众“舌尖上的安全”，积极创建国家食品安全示范市；切实加强安全生产监管，有效防范重特大安全事故，争创国家安全发展示范城市；深入推进平安建设，全力保障人民群众生命财产安全，着力创建全国平安建设示范区。加强民族宗教工作，促进团结和谐。认真做好国防动员工作，大力支持国防后备力量建设。

　　今年市政府将继续着力办好十件实事：

　　1、推进全民创业就业。支持1.5万人创业，开展就业培训6万人次，新增城镇就业人员10万人。

　　2、促进城乡教育资源均衡配置。启动建设襄阳“教育云”，全市中小学教育资源平台和管理服务平台覆盖面达到80%；完成150所农村寄宿制学校“五有”（宿舍、食堂、自来水、洗浴室、厕所）配套建设；资助家庭困难学生12万人次。

　　3、推进文体惠民。建设标准化乡镇（办事处）文化站（中心）20个，标准化村（社区）文化活动室100个、群众文化活动示范点100处，免费培训社会文化指导员和社会体育指导员各1000名；新建城乡健身点100处；向全市中小学生免费开放米公祠、绿影壁。

　　4、提高基层医疗卫生服务水平。新建和改建标准化村卫生室300家；新建和改建标准化社区卫生服务中心10家。

　　5、加强食品安全监管。改造升级菜市场10个，建设平价商店40家；建立肉菜流通追溯体系；建立市区餐厨废弃物无害化处理体系。

　　6、加快保障性住房建设。全市建设各类保障性住房5.2万套，其中公共租赁房7780套、棚户区改造3.5万套、其他保障性住房9342套；完成市区4个老旧住宅区综合整治。

　　7、关爱妇女儿童和老人。为2.9万对夫妻开展免费孕前优生健康检查；新建200所留守儿童爱心服务站；在城区建设60个社区居家养老服务中心，在农村建设40个老年人互助照料中心。

　　8、加强扶贫济困助残。向1.3万名特困残疾人发放生活补贴，改造农村危房4000户，实施扶贫搬迁700户，再解决20万农村人口饮水安全问题，为各类困难群体提供法律援助3500件以上。

　　9、完善城市基础设施。打通中心城区断头路3条，新建改建人行道20万平方米、新建人行过街通道5处，硬化、亮化背街小巷37条，综合整治“三无”小区100个，新建绿道40公里；启动建设南渠、大李沟（市区段）截污管网；新建改建港湾式公交站台20个，延伸、开通城市公交线路3条，开通快速公交线路1条，更新公交车辆200台。

　　10、建设美丽乡村。建设绿色示范乡村60个；新建改建农村综合交通服务站10座，新建农村候车亭100个、通村公路1000公里；新建改建村级综合服务社300家。

　　三、切实加强政府自身建设

　　新的一年，我们将以转变政府职能为重点，加强自身建设，努力创造良好发展环境、提供优质公共服务、维护社会公平正义。

　　（一）以解放思想为先导，着力建设创新型政府。我们将以自我革新的勇气和胸怀推动改革发展，牢固树立大局意识，冲破思想观念的障碍，跳出条条框框限制，克服部门利益掣肘；我们将以锐意进取的追求和担当推动改革发展，牢固树立责任意识，只要有利于襄阳的发展，只要有利于增进群众福祉，就义无反顾、勇往直前，大胆试、大胆闯，坚决破、坚决改；我们将以创新开放的措施和办法推进改革发展，牢固树立创新意识，尊重经济规律，坚持顶层设计，注重整体谋划，增强各项改革的关联性、系统性、可行性，用改革的办法、开放的思路和创新的举措破解发展难题，争创全省乃至全国改革示范试点。

　　（二）以提高效能为核心，着力建设服务型政府。以群众路线教育实践活动为契机，深入推进“假如我是服务对象”大讨论活动，促进各级政府和部门把群众的呼声、意愿和诉求作为施政理政的终极导向，把促进社会公平正义、增进人民福祉、回应人民期待作为推进改革发展的出发点和落脚点，面对面解决问题，实打实为民服务；切实提高为民服务效率，强化便民利企服务措施，深化政府绩效管理、精细化管理、专业化管理和规范化管理，以“钉钉子”的狠劲推进各项工作落实，下大力气解决市场主体和人民群众反映强烈的突出问题，切实增强政府公信力与执行力；切实提高为民服务能力，提高各级公务人员专业素养，提升各级政府和部门领导干部战略思维以及把握规律、科学决策和解决问题的能力。

　　（三）以依法行政为重点，着力建设法治政府。坚持依法决策，完善重大行政决策听证、合法性审查和风险评估等制度，健全政府法律顾问制度。强化依法行政考核和依法行政示范单位创建工作。加强行政执法监督，探索建立行政处罚案件信息公开制度，全面推行行政处罚案件法制机构审查、案审会集体讨论决定、重大行政处罚报备和行政执法案件评查机制，强化涉企行政执法电子监察。依法加强统计基础工作，继续做好第三次经济普查。自觉接受市人大的法律监督和市政协的民主监督，认真办理人大代表议案、建议和政协建议案、提案，认真听取民主党派、工商联、无党派人士和人民团体的意见建议。

　　（四）以落实制度为保障，着力建设廉洁政府。加强各级政府思想政治建设，认真落实党风廉政建设责任制，严格执行中央八项规定，贯彻厉行节约反对浪费条例，促进各级公务人员讲政治、守纪律。深入推进“四风”问题治理，建立健全深入基层、联系群众、调查研究常态化工作机制，下决心改进会风文风，推进部门预算和“三公”经费预算公开，严格控制“三公”经费支出。健全行政权力规范运行的长效机制，把权力关进制度的“笼子”；全面落实部门权力清单制度，让权力在阳光下运行；大力推行电子政务，充分利用政府网络平台推进政务公开、回应社会关切。加强行政监察和审计监督，严格实行重大项目行政审批、资金运作、施工操作隔离制度，完善公共资源配置、交易和管理，严肃查处重点领域的违纪违规问题，坚决纠正损害群众利益的各种不正之风。深入推进治庸问责、市民问政常态化，对不作为、慢作为、乱作为的部门和公务人员严格问责。

　　各位代表！当前襄阳正处于转型发展、跨越发展的关键时期，省委、省政府寄予厚望，全市人民殷切期待，我们重任在肩、使命光荣。我们将倍加珍惜时代赋予的机遇和平台，倍加珍惜全市人民的信任和支持，在市委的坚强领导下，坚持开拓进取，持续改革创新，努力谱写“两个中心、四个襄阳”建设新篇章！
